# MicroRNA-199: A Potential Therapeutic Tool for Hepatocellular Carcinoma in an Experimental Model

**DOI:** 10.31557/APJCP.2021.22.9.2771

**Published:** 2021-09

**Authors:** Shimaa Atta, Nabila El Kramani, Sara Reda Mohamed, Menna Adel Mohamed, Sara Hesham Hassan, Reem Hesham, Aya Mostafa Mohamed, Eman Eid Abdel-Halim, Yassmin Ashraf Mohamed, Eman El-Ahwany

**Affiliations:** *Immunology Lab, Theodor Bilharz Research Institute, Kornish El Nil street, Giza, Egypt. *

**Keywords:** HCC, MiRNA199a, caspase, VEGF, TNFα

## Abstract

Hepatocellular carcinoma is one of the major health problems throughout the world with a very poor prognosis. MicroRNAs are small regulatory non-protein-coding RNA molecules. We aimed at investigating microRNA-199 as a potential therapeutic tool for HCC both in vitro and in an experimental model. A therapeutic strategy based on the effect of microRNAs to target genes responsible for liver cancer was adopted in this work. The ability of these small RNAs to potently influence cellular behavior was also investigated. The role of miR-199a in the development of liver cancer has been identified using a systematic literature search using miRBase. HepG2 cell line was used to test the effect of miRNA199a in vitro. Hepatocellular carcinoma was induced in Male Balb/C mice by diethylnitrosamine (DEN). Mice were treated with miRNA-199a and sacrificed after 16 weeks and blood samples and liver specimens were collected for biochemical and histopathological assessment. Histopathological examination of liver specimens after miRNA 199a treatment showed regression of Hepatocellular carcinoma with restoration of normal architecture. AFP, VEGF and TNFα levels decreased after treatment with miRNA 199a. Caspase 3 and 9; showed decreased expression in animals treated with miRNA 199a than non-treated ones.

## Introduction

Hepatocellular carcinoma (HCC) is the 4^th^ cause of cancer-related death worldwide (Yang et al., 2019). HCC incidence and mortality are predicted to increase in African countries due to limited resources to combat high levels of viral infection and provactive environmental risk factors (Okeke et al., 2019). Egypt ranks the 3^rd^ and 15^th^ most populous country in Africa and worldwide, respectively (Rashed et al., 2020). The increasing incidence of HCC has led to an expanding interest in scientific research in this field. Therefore, a vast knowledge of experimental models that mimic the natural pathogenesis of HCC in a short time period is needed. HCC rodent models act as a critical bridge between laboratory-based research and human clinical studies (Collins et al., 2019). Animal models for HCC could be helpful to understand the molecular mechanisms underlying the pathogenesis of HCC, which may offer new insights for possible treatment options (Zhu et al., 2019). Due to various features, as the small size and the similarity to humans, the laboratory mouse remains one of the best models to study cancer in vivo (Kohn-Gaone et al., 2016). Therefore, the mouse model may open new prospects for early prediction of liver cancer and the development of new therapies for tumorigenesis in human.

The hepatocarcinogens are subdivided into two classes, namely genotoxic and non-genotoxic (or epigenetic) carcinogens (Heindryckx et al., 2009). The genotoxic carcinogens can presumably cause cancer by forming DNA adducts, which lead to genetic changes of the target cell. These changes can direct normal cells to a preneoplastic state (initiation) (Pascale et al., 2019). The non-genotoxic carcinogens do not modify DNA structure, but generally stimulate the preneoplastic or initiated cells to evolve into a malignant neoplasm by controlling cell proliferation, apoptosis and cell differentiation (Oliveira et al., 2007). DEN produces DNA-damage leading to genetic mutations in an otherwise healthy liver (Sanchez-Perez et al., 2005). It is typically administered to mice between 12 and 15 days of age by a single intraperitoneal injection (5 μg/g body weight) weekly (Teoh et al., 2008).

MicroRNAs (miRNAs) are small (19–25 nucleotides) regulatory non protein-coding RNA molecules. These RNAs serve as modulators of genes involved in various biological pathways, such as development, cell differentiation, cell proliferation, cell death, chromosome modifications, virus pathogenesis, and oncogenesis (Gebert and MacRae, 2019). Currently, miRNAs play fundamentally important roles in regulation of gene expression in many organisms by acting on messenger RNA (mRNA) at post-transcriptional levels (Bartel, 2018). Many miRNAs have subsequently been found to have links with various types of cancer and accordingly are sometimes referred to as “oncomirs” (Fang et al., 2017). miR-199 family consists of miR-199a and miR-199b (Jiang et al., 2017). Many studies have demonstrated that mature miR-199a is a key player in the maintenance of normal homeostasis and in the regulation of disease pathogenesis (Song et al., 2010; Alexander et al., 2013; Lino Cardenas et al., 2013; Hsu et al., 2014; Li et al., 2016; An et al., 2017). Hou et al., (2011) found that miR-199a/b-3p can act as a tumor suppressor that inhibits HCC growth.

## Materials and Methods

*Bioinformatics*

We used the Bioinformatics server of miRNA-target prediction (miRBase) (http://www.mirbase.org/index.shtml), which aims to provide integrated interfaces to comprehensive microRNA sequence data, annotation and predicted gene targets. Specific target genes of miR-199a in hepatocellular carcinoma (HCC) were identified by using this server. First, we searched for our target miRNA-199. Then, we selected hsa-mir-199a which referred to Homo sapiens miRna-199a. Hsa-mir-199a has two mature sequences, hsa-miR-199a-5p and hsa-miR-199a-3p, each mature mir-199a contains a number of target genes and links that were provided to “predicted target” pages. The target genes which are regulated either by hsa-miR-199a-5p or hsa-miR-199a-3p were determined by using (miRDB) database (http://www.mirdb.org/index.html). The target scores for each gene and their sequences from (GenBank) were also identified. Generally, there were 562 predicted targets for hsa-miR-199a-5p and 477 predicted targets for hsa-miR-199a-3p in miRDB. Our analysis showed 14 comprehensive potential target genes for hsa-miR-199a-3p and hsa-miR-199a-5p which have been related to HCC. 

*The Selection of microRNAs*

The role of miR-199 in the development of liver cancer especially HCC has been identified using a systematic literature search. The search was conducted in the electronic databases PubMed (https://www.ncbi.nlm.nih.gov/pubmed) and Google Scholar (https://scholar.google.com) until November 2019. The following key words were used: ‘Malignant OR cancer OR tumor OR neoplasm OR carcinoma,’ ‘hepatocellular OR liver OR hepatic OR HCC’ and ‘miR-199 OR miRNA-199 OR microRNA-199 OR miR 199 OR miRNA 199 OR microRNA 199.’

*In vitro study*

*Cell Culture*

Human hepatocellular carcinoma HepG2 cell line (provided from Immunology lab, TBRI) was revived and cultured in 75 cm^2^ culture flasks (Greiner bio-one GmbH, Germany) containing Roswell Park Memorial Institute (RPMI) 1640 Medium (Sigma Aldrich, Germany) supplemented with 100 ml/L fetal bovine serum (FBS), 10 g/L L-glutamine (Bio Whittaker, a Combrex Company, Belgium) and 10 g/L penicillin/streptomycin. HepG2 cells were cultured at 37^o^C in a 5% CO_2_ incubator for 48 hours (hrs) till full confluency, cells were dissociated from the culture flask by 0.025% trypsin (Sigma) and the culture flask was gently shaken to ensure that a single-cell suspension was obtained. Cells were subcultured till reaching the required cell count. The cells were counted using a Neubauer hemocytometer and viability was tested by the trypan blue dye exclusion assay using trypan blue (5 g/L; Biochrom KG, Berlin, Germany).

*Neutral Red Cytotoxicity Assay*

After trypsinization, cells were seeded in a 96-well microtitre plate (Corning) at a concentration of 5×10^4^ cell/well. The plates were incubated at 37^o^C in 5% CO_2_ for 24 hrs till confluency. Culture medium containing different concentrations of mir 199 (15, 30, 60, 125, 250, 500, 1000 and 2000 µg/mL), was added in triplicate.cells only were used as untreated control. Plates were incubated for 72 hrs. Culture media were discarded and 50µl of dye medium (1ml Neutral red stock diluted in 10ml RPMI) were added to each well and incubated for 2 hrs. The dye-medium was removed and the plates were washed with PBS, pH = 7.4 three times. 50 μL of acetic acid-ethanol (one ml glacial acetic acid in 100 ml 50% ethanol) were added and the plates were kept on a plate shaker for 10 min at room temperature to extract the dye. The absorbance of the extracted dye was measured by spectrophotometric reading (Spectra max 190 Molecular devices) using 540 nm filter. The mean of three measurements for each concentration was determined.

Calculation: The mean ± SEM of three separate experiments for each concentration was determined.

The inhibition percentage = (A*control- A dose/ A control -A blank) ×100

A* is the absorbance at wave length 490-520 nm, Control = Untreated cells, Blank = Media without cells.

*Calculations of IC50*

Dose-response curves were plotted, and 50% inhibitory concentrations of miRNA (IC_50_) were calculated through GraphPad Prism 8 program and Microsoft Excel 2010 program. Experiments were carried out in triplicates and the data were presented as mean ± SEM.

*In vivo study*

*Animals*

Two weeks old male Balb/C mice were provided from the animal house, Theodor Bilharz Research Institute (TBRI), Giza, Egypt. Animals were maintained less than 12h light/dark cycles, fed on a standard diet and given free access to water. All experimental procedures applied on animals were applied according to guidelines for the care and use of laboratory animals, National Research Council, USA.

*Induction of hepatocellular carcinoma*

Tumors were induced in male mice by a single intraperitoneal (i.p.) injection of DEN (Sigma, Aldrich) diluted in phosphate buffer saline (PBS) weekly for 16 weeks at a dose of 50 mg/kg body weight using a 29G syringe producing DNA damage that led to cellular changes. 

*Design of miRNA-199a*

MiRNA-199a was manufactured as the following sequence of miR-199a precursor:

CCCAGUGUUCAGACUACCUGUUC

*Animal study*

Thirty male mice were categorized into three main groups (10 mice / each); Normal control group: were injected i.p. with PBS as a vehicle control; mice in HCC Pathological group received i.p. injection with DEN; mice in miRNA-199a treated group were injected intrahepatically only once with100 μl of miRNA-199a per animal after one week post-injection of DEN. After 16 weeks post-injection of DEN, animals of each group were euthanized. Retro-orbital sampling was performed in mice under transient anesthesia using isoflurane by penetrating the retro-orbital sinus with a glass capillary. Blood samples were collected in tubes and centrifuged at 2000 xg for 20 min. Serum was transferred into a fresh tube and stored at -20 °C for further analysis. After complete death under cervical dislocation, mice were dissected to harvest livers that were washed with PBS, and immediately fixed in 10% buffered formalin for histological evaluation. 

*Histological examination*

Fixed liver tissues were dehydrated in alcohol with growing concentrations: 70% - 85% - 90%, inhibiting tissue damage and water is finally removed by absolute alcohol baths. Then tissues were embedded in hot paraffin. The paraffin blocks were sectioned at 4-5 μm thickness. Then, the sections were deparaffinized and dehydrated through grade ethanol dilutions and finally stained with hematoxylin and eosin staining. The sections were examined under light microscope. 

*Measurement of tumor markers*

Serum concentration of Alfa-Fetoprotein (AFP) was determined by Quantikine® ELISA kits (R&D Systems, Inc., USA). 

*Estimation of vascular endothelial growth factor and tumor necrosis factor alfa*

Quantitative determination of mouse vascular endothelial growth factor (VEGF) and tumor necrosis factor alfa (TNF α) concentrations in serum sample was done by Quantikine^®^ELISA kit (R&D Systems, Inc., Minneapolis, USA). The steps were performed according to the manufacture’ instructions. Optical density was measured at 450 nm.

*Estimation of apoptotic markers*

Caspase-3 and 9 were estimated by Real time Polymerase chain reaction

*RNA extraction and cDNA synthesis*

Liver tissue specimens were collected and stored directly at − 80°C for RNA preservation. Total RNA was extracted using Qiagen RNeasy extraction kit (cat no: 74104) according to manufacturer’s kit instructions. RNA concentration was quantified using NanoDrop2000 Spectrophotometer. Reverse transcription was then performed to obtain the cDNA corresponding to the mRNA isolated from the hepatic specimens. cDNA synthesis was conducted using the applied biosystems high capacity cDNA reverse transcription kit. Each reaction consisted of 2µl reverse transcriptase buffer, 0.8µl dNTPs, 2µl random primer, 1µl reverse transcriptase enzyme, 1µl RNase Inhibitor, 1µg of total RNA and completed with RNase-free water to form a total reaction of 20µl. cDNA was obtained using Biometra T Professional Thermocycler (Germany), under; 25°C for 10 min, 37°C for 120 min, 85°C for 5 min and finally reaction stopped at 4°C.

*Quantitative reverse transcription real time PCR (qRT-PCR)*

Amplification of cDNA was performed using SYBR Green I (Thermo Scientific) and Real Time (RT)- PCR 7500 (Life Technologies, Applied Biosystems, Foster City, CA, USA). For endogenous control, β-actin was used in each experiment as the housekeeping gene for normalization. Sequence of caspase-9 forward primer was F-5’-GCTGTGTCAAGTTTGCCTACCC-3’ and caspase-9 reverse primer sequence was R-5’-CCAGAATGCCATCCAAGGTCTC-3’ β-actin forward primer sequence was F-5’-GGCATCCTGACCCTGAAGTA-3’ and β-actin reverse primer sequence was R-5’-GGGGTGTTGAAGGTCTCAAA-3’. One tube was used for caspase-9 and another for β-actin. For each qRT-PCR reaction, 2µl of the cDNA template products, 12.5 µl RT2 SYBR Green ROX qPCR master mix (cat no: 330520, QIAGEN), 1µl of each of the forward and reverse primers, 8.5 µl of RNAase free water, in a final volume of 25 µl reaction were mixed. Cycling conditions were; initial denaturation step at 95°C for 10 minutes, followed by 45 cycles, each consisting of; denaturation at 95°C for 15sec, annealing step at 60°C for 1 min, and a final extension step at 60°C for 10 min. Melting curve conditions are started right after qRT-PCR completion and are as follows; 95°C for 15 sec, 60°C for 1 min and 95°C for 15 sec. Relative expression of these markers was quantified by using of comparative threshold cycle (CT) method.

*Statistical analysis*

One Way ANOVA (parametric) was used to test the effect of experimental periods of the studied parameter. Duncan’s test to homogeneity was used to compare between each two dependent variables. Data were represented as a mean of 10 mice ± SEM. Data were exhibited a significant effect or difference at α=0.001 and α=0.05. The statistical analysis was done by the aid of Statistical Package for the Social Sciences (SPSS) version 24.6. 

## Results

*Bioinformatics*

**Figure 1:**
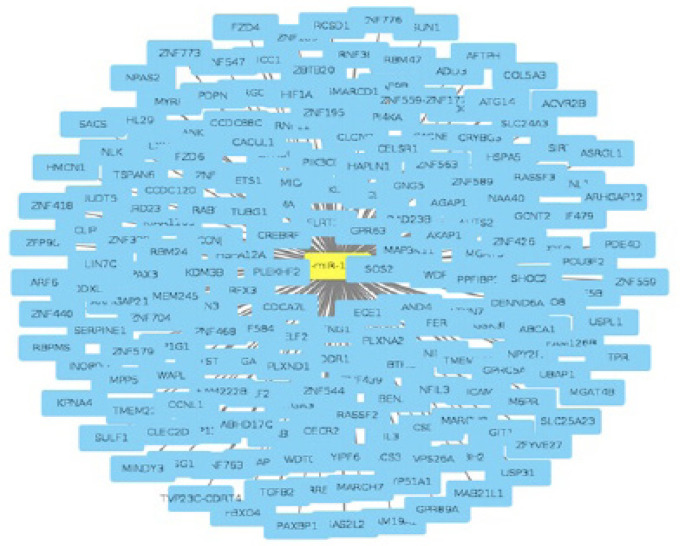
Target Genes of miR-199 Using Cytoscape Software

According to the steps we had discussed previously, we found out that miRNA 199a has more than 400 target genes in the body that reacts differently according to the location of miRNA in the body. Then, we shortlisted our predicted target genes to those with prediction score > 80, as these genes represent the most confident and highly reliable targets. Our shortlist included 162 predicted target genes for hsa-miR-199a-3p and 189 predicted target genes for hsa-miR-199a-5p, from which only 14 genes were found to be related to HCC. We used Cytoscape (https://cytoscape.org) (Figure 1), which is an open source software platform for visualizing complex networks and integrating these with any type of attribute data, to visualize miRNA 199 and its predicted target genes. 

*In vitro study*

*Neutral Red Cytotoxicity Assay*

Inhibition effect of the miRNA199a on HepG2 cells was evaluated using the neutral red cytotoxicity assay. The proliferation of HepG2 cells was inhibited on using miRNA199a in a concentration-dependent manner. 

**Figure 2:**
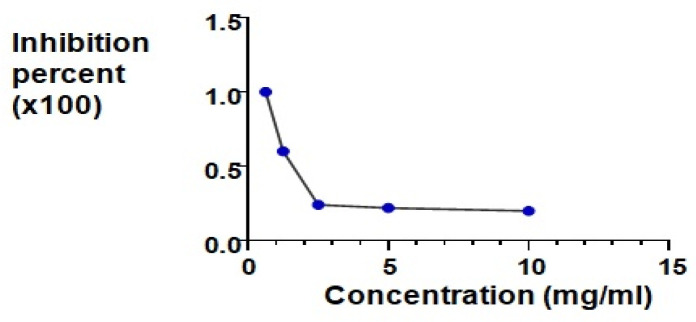
The Inhibition of Proliferation Activity in the HepG2 Cell Line of miRNA 199 Using the Nneutral Red Cytotoxic Assay

Cells were evaluated by morphological changes; wells treated with miRNA199a showed disturbance of the cell monolayer as well as characteristic changes of cell death including granulation, blebbing, shrinkage and nuclear fragmentation. miRNA199a was active in the inhibition of proliferation of HepG2 cells as proved by calculating inhibition activity (%) using graphpad prism 8. IC_50_ was found to be (0.05±0.0025) mg/mL (Figure 2).

*In vivo study*

*Histopathologic Examination of Liver Specimens*

**Figure 3:**
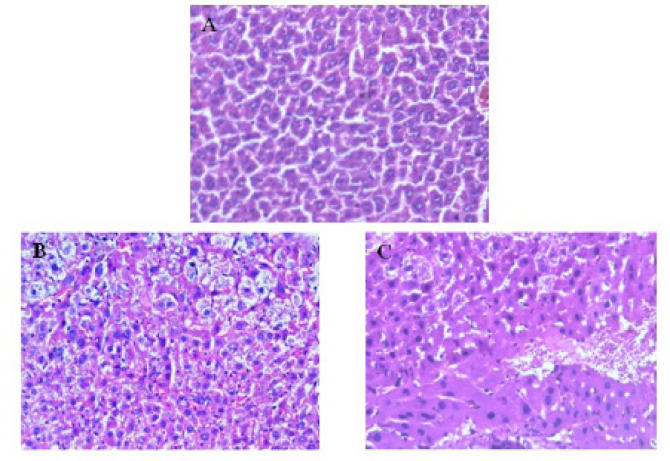
Liver Specimens of Different Groups. A, normal liver; B, Pathological control (DEN injected group) shows loss of normal architecture with multiple mitotic figures, increased nucleocytoplasmic ratio and sever ballooning and hydropic degeneration; C, treated group (mir199 treated group) shows restoration of normal architecture, decreased mitotic figures and minimal ballooning of hepatocytes with inflammatory infiltrate

Microscopic examination of liver sections of mice after DEN injection showed loss of hepatic lobular architecture with severe hydropic degeneration of the hepatocytes. Portal tracts were thickened and extended with chronic inflammatory cells and fibrotic tissue. There was also marked polymorphism, increase in mitotic activity and increase in nuclear size as illustrated in Figure 3B.

After treatment with miRNA-199a, liver specimens showed restoration of normal liver architecture, moderate inflammatory infiltrate, decreased mitotic activity, decreased nuclear size, mild polymorphism compared to the pathological non-treated group (Figure 3C).

*Estimation of tumor markers*

*AFP*

**Figure 4:**
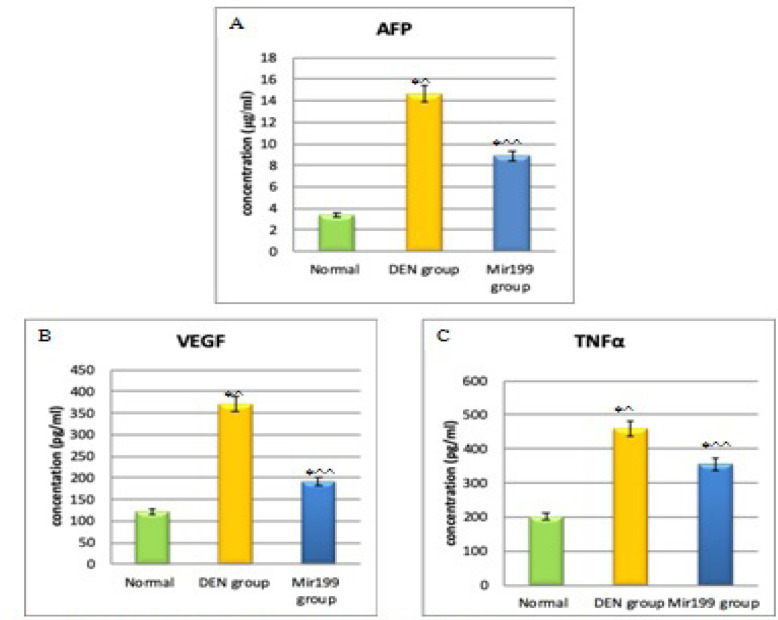
Levels of (A) AFP, (B) VEGF and (C) TNFα in Different Test Groups. *P ˂0.001, very high significance, ^, increase compared to normal group; ^^, decrease compared to DEN group

Our results revealed that AFP level in DEN treated mice serum samples was found to be 14.63±2.32 ng/ml which is higher than normal control (3.38±.64 ng/ml) with very high significance (P ˂0.001). While there was very high significant decrease in AFP levels in sera of mice treated with mir199 to reach 8.92±.57 ng/ml when compared to mice injected with DEN (P ˂0.001) (Figure 4A). 

*Estimation of vascular endothelial growth factor and tumor necrosis factor alfa*

*VEGF*

The results of this study revealed that VEGF level in DEN treated mice serum samples is 371.17±9.64 pg/ml that is higher than normal mice (121.47±3.34 pg/ml) with very high significance (P ˂0.001). While after treatment with mir199, VEGF level decreased markedly (191.56±6.07 pg/ml) with very high significance compared to mice injected with DEN (P ˂0.001) (Figure 4B).

*TNFα*

Also there was an elevation in TNFα level (459.27±7.49 pg/ml) with very high significance after injection with DEN when compared to level in normal mice sera (200.37±8.71 pg/ml) (P ˂0.001). With mir199 treatment, there was a very high significant decrease in level of TNFα (355.41±8.29 pg/ml) in comparison to mice injected with DEN (P ˂0.001) (Figure 4C).

*Estimation of apoptotic markers*

*Caspase 3*

**Figure 5:**
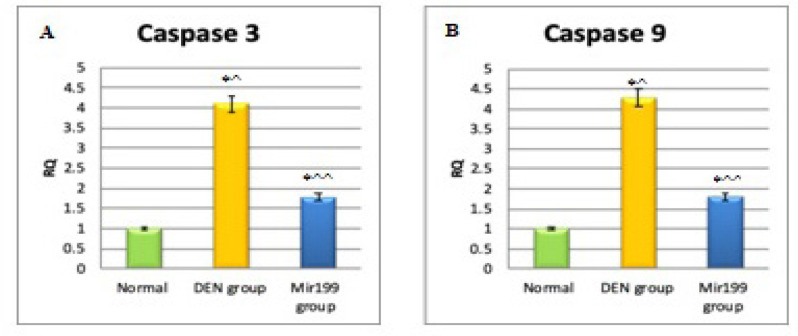
Relative Quantification Values of (A) Caspase-3 and (B) Caspase 9 in DEN and miRNA-199-Treated Groups. *P ˂0.001, very high significance; ^, increase compared to normal group; ^^, decrease compared to DEN group

Relative quantification value of Caspase 3 was found to be elevated by 4.1 folds after injection with DEN when compared to its value in normal mice sera with very high significance (P ˂0.001). Our data also showed that there was a very high significant decrease in Relative quantification value of Caspase 3 in miR199 treated mice sera in comparison to mice injected with DEN (P ˂0.001) (Figure 5A). 

*Caspase 9*

Our results showed that Relative quantification value of Caspase 9 in DEN injected mice was higher than its level in normal mice sera by 4.28 folds with very high significance (P ˂0.001). After treatment with miR199a, there was a very high significant decrease in relative quantification value of caspase 9 in comparison to mice injected only with DEN (P ˂0.001) (Figure 5B). 

## Discussion

Hepatocellular carcinoma (HCC) is one of the most common primary malignant tumors in the world and the third leading cause of cancer-related death. It accounts ˃ 90% of primary liver malignancies (Bray, 2018; Forner et al., 2018). Treatment lines of HCC include surgery, liver transplantation, ablative therapies, trans-arterial embolization, radiotherapy, and chemotherapy and immunotherapy (Abdalla and Stuart, 2019). New approaches for treatment HCC are being investigated including microRNAs, Golgi-73 Protein (GP73), Glypican-3 (GPC3), Osteopontin (OPN), and more (Tunissiolli et al., 2017).

MiRNAs are conservative noncoding RNA, of around 19~25 nucleotides in length (Berindan-Neagoe et al., 2014; Gebert and MacRae, 2019), It is responsible for mRNA degradation or inhibiting its transcription (Rupaimoole and Slack, 2017; Ha and Kim, 2019). This may play a role in the mechanism of tumor formation (Giordano and Columbano, 2013; Berindan-Neagoe et al., 2014; Frampton et al., 2015) or tumor suppression through blocking cell cycle, increasing apoptosis, and reducing tumor angiogenesis and metastasis by inhibiting migration and invasion (Murakami et al., 2006; Hou et al., 2011).

In our study, we aimed to investigate microRNA-199 as a potential therapeutic tool for HCC both *in vitro* and in an experimental model. 

The role of miR-199a in the development of liver cancer has been identified using a systematic literature search. Specific target genes of miR-199a in hepatocellular carcinoma were identified using various online bioinformatics tools. We reveal the functionality of this micro-RNA through target prediction and functional annotations. In the current study, in silico analysis using novel servers (miRbase, miRDB) has demonstrated the individual target genes of miR-199a and further investigated its function in HCC progression.

Due to the high cost of isolation and culture, limited availability, short life span, metabolic and genetic differences, loss of functions and hepatic phenotype in two-dimensional culture systems; human hepatocytes are not used for *in vitro* cytotoxicity studies (Štampar et al., 2019). 

HepG2 cells are immortalized cell line consisting of human well-differentiated hepatocellular carcinoma cells of a 15-year-old Caucasian male. It is an ideal *in vitro* model (Nikolic et al., 2018).

The functional characteristics of HepG2 cells is their ability to express many differentiated hepatic functions, such as synthesis and secretion of plasma proteins, cholesterol and triglyceride metabolism, lipoprotein metabolism and transport, bile acid synthesis, glycogen synthesis, or insulin signaling (Donato et al., 2014).

In our study, HepG2 cell line was used to test the effect of miRNA199a *in vitro*. Cells were cultured in increasing concentrations of miRNA 199a and cell viability was tested using neutral red assay. Mir199a was found to be active in the inhibition of proliferation of HepG2 cells. This came in agreement with Gui et al. (2016) who declared that forced expression of miR-199a-5p by a lentiviral vector expressing miR-199a-5p inhibited HepG2 cell proliferation as elucidated by decreased expression of Proliferating cell nuclear antigen (PCNA), a marker of tumor cell proliferation (Gui et al., 2016). Similar results were presented by Guo et al., (2017). It was reported that miR-199a inhibits the proliferation of hepatoma cells by targeting hypoxia-inducible factor-1a and cluster of differentiation 44, as well as by regulating the cell cycle. In addition, miR199a upregulates CDKNlB/p27 and CDKN1A/p21 to inhibit the progression of the cell cycle, thereby inducing apoptosis (Fornari et al., 2010).

Many chemical compounds are used to induce carcinoma. The first chemically induced model of HCC was developed by the Japanese researcher, Riojun Kinosita. Since then, chemically induced animal models have been widely used (Santos et al., 2017). The advantage of chemically induced models is the similarity with the injury-fibrosis-malignancy cycle (Heindryckx et al., 2009). DEN is the most widely used genotoxic agent for in vivo chemically induced HCC (Connor et al., 2018). Tolba et al., (2015) stated that the most commonly used route is the intraperitoneal injection of DEN solution, however other routes such as intravenous, oral administration with drinking water or diet, inhalation, or intratracheal or intragastric instillation may be used (Tolba et al., 2015).

In our study, hepatocellular carcinoma developed in male Balb/C mice after 16 weeks of single intraperitoneal DEN injection/week. Several researchers also used DEN to induce HCC in animal models (Schiffer et al., 2005; Uehara et al., 2014; Roth et al., 2017; Jilkova et al., 2018).

Expression of miRNA 199a was found to be decreased among patients with HCC as revealed by Zhang et al., (2014) and Kamel et al., (2016). Hou et al., (2011) who used next generation sequencing to investigate the miRNomes in normal liver, liver with viral hepatitis, as well as liver cancer declared that in HCC miR-199a is lowered and this is accompanied with bad prognosis. Moreover, both *in vitro* and i*n vivo*, the PAK4/Raf/MEK/ERK pathway is inhibited by miRNA-199a targeting tumor-promoting PAK4 to suppress HCC growth. MiRNA-99a was also found to directly target insulin-like growth factor 1 receptor (IGF-1R) and mTOR, suggesting that miR-99a is a promising tumor suppressor for HCC (Li et al., 2011).

Thus we assumed that treatment of HCC with miRNA 199a could improve both liver structure and function. Our results showed that after treatment with miRNA 199a, histopathological examination of liver specimens of mice liver showed restoration of normal liver architecture, decreased mitotic activity, decreased nuclear size, decreased polymorphic figures. This came in agreement with Hou et al., (2011) and Li et al., (2011). 

In our study, AFP showed improvement with treatment with miRNA199a. Kamel et al., (2016) also showed that serum miR-199a was inversely correlated to AFP in patients with HCC. Our data state that VEGF level decreases with treatment with miRNA 199a. This came in agreement with Ghosh et al., who reported that miRNA199a could suppresses tumor growth and migration of HCC through inhibiting angiogenesis in by targeting VEGFA, and subsequently inhibiting VEGF secretion (Ghosh et al., 2017).

In this study, TNFα was also found to decrease in response to miRNA 199a treatment. This is in agreement with Zhang et al., (2019) who reported that MiR-199a has a negative association with TNF-a expression both *in vivo* and *in vitro*. 

Cell proliferation is counterbalanced by apoptosis. Some miRNAs influence cancer development by downregulating apoptosis. This was approved by Jovanovic and Hengartner, (2006). We found that Caspase3 and 9 expressions were lower with treatment with miRNA199a indicating decreased apoptosis. These findings were in agreement with kamel et al., (2016) who reported that apoptotic markers are inversely proportion to miRNA199a level. Our results were against results described by Chu et al., 2014 and Yan et al., 2018 who reported that MicroRNA 199a-5p increases apoptosis. 

In conclusion, miRNA 199a treatment with a single intrahepatic injection could improve both histopathologic structure and decrease AFP, VEGF and TNFα levels in HCC animal model. MiRNA 199a decreased apoptosis as proved by lowering expression of caspase 3 and 9.

## Author Contribution Statement

Conceptualization: Eman El-Ahwany. Methodology: Eman El-Ahwany. Software: Nabila El Kramani, Sara Hesham Hassan, Menna Adel Mohamed, Sara Reda Mohamed, Reem Hesham, Aya Mostafa Mohamed, Eman Eid Abdel-Halim, Yassmin Ashraf Mohamed. Validation: Eman El-Ahwany , Shimaa Atta. Formal Analysis: Shimaa Atta. Investigation: Shimaa Atta, Sara Hesham Hassan, Menna Adel Mohamed, Sara Reda Mohamed, Reem Hesham, Aya Mostafa Mohamed, Eman Eid Abdel-Halim, Yassmin Ashraf Mohamed. Resources: Shimaa Atta. Writing – Original Draft: Shimaa Atta. Writing – Review & Editing: Shimaa Atta, Eman El-Ahwany, Nabila El Kramani. Visualization: Shimaa Atta, Eman El-Ahwany. Supervision: Shimaa Atta, Eman El-Ahwany. Project Administration: Eman El-Ahwany. Funding Acquisition: Eman El-Ahwany
